# Corneal reshaping: an experiment with a type I collagen-based vitrigel for remodeling porcine corneas

**DOI:** 10.5935/0004-2749.2022-0128

**Published:** 2023-03-08

**Authors:** Maria Carolina Marquezan, Denise de Freitas, Shoumyo Majumdar, Xiaokun Wang, Jennifer Elisseeff, David L. Guyton, Kraig Scot Bower, Zachary P. Skurski, Maria Regina Chalita, Rubens Belfort Jr, Albert S. Jun

**Affiliations:** 1 Wilmer Eye Institute, Johns Hopkins University School of Medicine, Baltimore, Maryland, USA; 2 Department of Ophthalmology and Visual Sciences, Escola Paulista de Medicina, Universidade Federal de São Paulo, São Paulo, SP, Brazil; 3 Translational Tissue Engineering Center, Johns Hopkins University, Baltimore, Maryland, USA; 4 Department of Ophthalmology, Hospital Universitário de Brasília, Universidade de Brasília, Brasília, DF, Brazil

**Keywords:** Cornea, Corneal surgery, laser, Corneal topography, Corneal stroma, Prostheses and implants, Lasers excimer, Biocompatible materials, Animals, Swine, Córnea, Cirurgia da córnea a laser, Substância própria, Proteses e implantes, Lasers de excimer, Materiais bio-compatíveis, Animais, Suínos

## Abstract

**Purpose:**

This study aimed to report an experiment designed to determine anatomical changes in porcine corneas following placement of a novel polymer implant into the cornea.

**Methods:**

An *ex vivo* porcine eye model was used. A novel type I collagen-based vitrigel implant (6 mm in diameter) was shaped with an excimer laser on the posterior surface to create three planoconcave shapes. Implants were inserted into a manually dissected stromal pocket at a depth of approximately 200 µm. Three treatment groups were defined: group A (n=3), maximal ablation depth 70 µm; Group B (n=3), maximal ablation depth 64 µm; and group C (n=3), maximal ablation depth 104 µm, with a central hole. A control group (D, n=3) was included, in which a stromal pocket was created but biomaterial was not inserted. Eyes were evaluated by optical coherence tomography (OCT) and corneal tomography.

**Results:**

Corneal tomography showed a trend for a decreased mean keratometry in all four groups. Optical coherence tomography showed corneas with implants placed within the anterior stroma and visible flattening, whereas the corneas in the control group did not qualitatively change shape.

**Conclusions:**

The novel planoconcave biomaterial implant described herein could reshape the cornea in an ex vivo model, resulting in the flattening of the cornea. Further studies are needed using in vivo animal models to confirm such findings.

## Abstract (pt)

**Objetivo:**

Relatar um experimento projetado para determinar alterações anatômicas em córneas porcinas após a colocação de um novo implante de polímero na córnea.

**Métodos:**

Foi utilizado olho de porco *ex vivo*. Um novo agente modelador biocompatível, de colágeno tipo 1, com 6mm de diâmetro foi moldado com excimer laser em sua face posterior, para criar três formatos planocôncavos. Os implantes foram inseridos dentro de um bolsão, dissecado manualmente, a 200 micrômetros (µm). Foram definidos três grupos de tratamento: grupo A (n=3), teve a profundidade máxima de ablação de 70 µm; o grupo B (n=3), profundidade máxima de ablação de 64 µm; e o grupo C (n=3), profundidade máxima de ablação de 104 µm, com buraco central. O grupo controle, D (n=3), foi incluído, com a criação do bolsão estromal, porém sem inserir o material. A avaliação desses olhos foi realizada por tomografia de coerência óptica (OCT) e por tomografia corneana.

**Resultados:**

A tomografia corneana mostrou uma tendência para diminuição da ceratometria média em todos os 4 grupos. A tomografia de coerência óptica mostrou córneas com implantes localizados no estroma anterior e aplanamento visível, enquanto as córneas não mudaram qualitativamente o formato no grupo controle.

**Conclusões:**

O novo implante de biomaterial planocôncavo descrito aqui foi capaz de remodelar a córnea em modelo de animal *ex vivo,* resultando no aplanamento corneano. Novos estudos são necessários usando modelos animais *in vivo* para confirmar tais achados.

## INTRODUCTION

Corneal inlays of synthetic or biological materials to alter corneal shape have been described with variable successes^(1^-^3)^. This concept was introduced by Barraquer as synthetic keratophakia using glass^(4)^. Subsequently, the use of corneal tissues and other synthetic materials with higher permeability, as well as implant shaping using an excimer or a femtosecond laser, has been described^(4^-^17)^.

From the aforementioned experiments, Barraquer^(4)^ concluded the following: [1] The intracorneal inclusion of lenticules of foreign materials results ocular refraction modification, but is poorly tolerated by the cornea. [2] The inclusion of lenticules in human corneal tissues within the lamellae of the cornea results in a permanent and stable modification of the dioptric power of the cornea, with good tolerance and definitive incorporation of the lenticule. [3] This modification depends mainly on the change in the radius of the curvature of the anterior face of the cornea produced by the lenticule. [4] The modification of refraction is related to the shape (power) of the lenticule and its depth of placement within the corneal parenchyma.

Reshaping the cornea by adding an intrastromal biomaterial has been an attractive prospect because it is not based on removing tissue, does not involve concerns about corneal thickness and iatrogenic cornea ectasial risk as current laser treatments do, and bypasses the risk and cost issues of phakic intraocular lenses with attendant risks of intraocular surgery. Current models and materials of corneal implants have made corneal inlays a promising alternative for the treatment of presbyopia by altering the curvature and/or optical properties of the corneal surface through small incisions^(18)^. The main issue is to find the optimal material; thus, human tissue is not needed. This study aimed to investigate whether a new biocompatible polymer implant shaped with an excimer laser can alter the curvature of the cornea.

## METHODS

### Biomaterial preparation

Type I bovine collagen solution (Cosmo Bio Co., LTD., Tokyo, Japan) was dispensed into plastic molds. Solutions were neutralized by ammonia exposure at room temperature and incubated for 30 min at 37°C to complete gelation. Samples were then vitrified in a 39°C vitrification chamber at 40% relative humidity for 3 days. Following vitrification, biomaterials were rehydrated and chemically crosslinked using 3 mg/mL 1-ethyl-3-(3-dimethylaminopropyl)-carbodiimide and 3 mg/mL N-hydroxysuccinimide for 30 min. Before further use, the implants were washed several times in phosphate-buffered solution. The lenticule was prepared by the bioengineer group in accordance to published literature^(19^-^21)^.

### Shaping of the implant material

The vitrigel starting material was approximately 240 µm thick, 6 mm in diameter, and flat on both anterior and posterior surfaces. The material was treated with excimer laser (VISX STAR S4 IR^®^ Excimer Laser System software version 5.3, Santa Clara, CA, USA) using a multizone phototherapeutic keratectomy (PTK) treatment on the posterior side to create a planoconcave shape.

To achieve the desired concave shape and dioptric power of the implant, treatments were determined in the following manner: sequential treatments on the same tissue for groups A, B, and C are specified below, ranging from 5 mm to 2 mm in diameter and from 4 µm to 24 µm in thickness:

Group A: 5.0 mm/8 µm ➪ 4.0 mm/12 µm ➪ 3.5 mm/ 16 µm ➪ 3.0 mm/20 µm ➪ 2.5 mm/8 µm ➪ 2.0 mm/6 µm

Group B: 5.0 mm/8 µm ➪ 4.0 mm/12 µm ➪ 3.5 mm/ 16 µm ➪ 3.0 mm/ 18 µm ➪ 2.5 mm/6 µm ➪ 2.0 mm/4 µm.

Group C: 5.0 mm/12 µm ➪ 4.0 mm/16 µm ➪ 3.5 mm/ 20 µm ➪ 3.0 mm/24 µm ➪ 2.5 mm/16 µm ➪ 2.0 mm/16 µm.

Group D: control group, including a stromal pocket without biomaterial insertion.

This planoconcave shape was intended to optimally flatten the cornea following Barraquer’s theory^(4)^. Diopter (D) correction was estimated according to the Munnerlyn formula, which states that the thickness of the tissue ablated in microns (t) is equal to the square of the diameter of the optical ablation zone in millimeters (S) multiplied by diopter correction (D) divided by 3 (t=S^2^D/3)^(22)^. Excimer laser-treated implants were stored dry until subsequent use.

### Surgical procedure

In total, 12 fresh porcine eyes with intact epithelia and clear corneas were retrieved from the local slaugh-terhouse within 24 h postmortem. An arbitrary mark was made on each cornea using a surgical marking pen and considered the 12 o’clock position reference from this point onwards, which was used to position each globe for subsequent analyses. Balanced salt solution was injected into the posterior segment to achieve physiologic tension by digital palpation for all subsequent measurements and imaging.

Then, the eyes were attached to a styrofoam base with needles. A 7-mm straight superior incision was made at the limbus, using a 200-µm guarded depth blade (Rubenstein Preset LRI Lancet Diamond Knive, MicroSurgical Technology^TM^, Malvern, PA, USA). At this depth, a 9-mm stromal pocket centered over the pupil was made using a Crescent Knife ClearCut^®^ (Alcon Canada, Mississauga, ON, Canada) and a Morlet Lamellar Dissector and Knife Dissector (Duckworth & Kent Ltd., Hertfordshire, UK). Biomaterials from groups A-C were centered in the stromal pocket. No suture was placed after the procedure. Intrastromal implantation was performed 1 day after excimer treatment of the lenticule.

### Evaluation of porcine eyes by Visante^®^ and Pentacam^®^

Baseline values were measured by corneal tomography (Pentacam^®^, Oculus, Wetzlar, Germany) and anterior-segment optical coherence tomography (AS-OCT, Visante^®^, Carl Zeiss Meditec, Dublin, CA, USA) after the completion of the corneal pockets, but before implant insertion. Post-implantation measurements were performed in the same manner within 1-2 h of implantation of the designated biomaterial. In the control group, imaging was performed before and after completion of the stromal pockets. OCT measurements were obtained with a caliper from the anterior to the posterior aspect of the lenticule. AS-OCT images were used to assess corneal thickness, lenticule thickness, and pocket depth for each eye.

### Statistics

The Kruskal-Wallis test was performed to evaluate the differences in keratometry among the four groups. The Wilcoxon matched-pair signed-rank test was used to evaluate the differences in keratometry before and after intracorneal lenticule implantation within each group. The Wilcoxon rank-sum test (also known as the Mann-Whitney two-sample statistics) was performed when evaluating the differences in keratometry between group D and the other three groups together (Stata/SE 12.0, StataCorp LLC, College Station, TX, USA). P-values of <0.05 were considered statistically significant.

## RESULTS

Six experiments were performed, improving the lenticule quality, intrastromal implantation technique, and evaluation. Data were not published.

**Figure 1:**
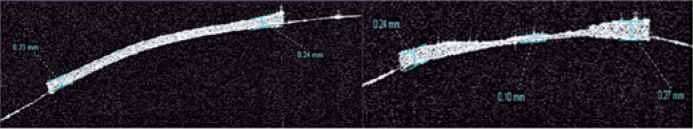
Visante® anterior-segment OCT of the implant from group A, before (left) and after (right) excimer laser treatment.

Excimer laser treatment of the biomaterials resulted in a central concavity as shown by OCT in a representative sample from group A (Figure 1).

**Figure 2:**
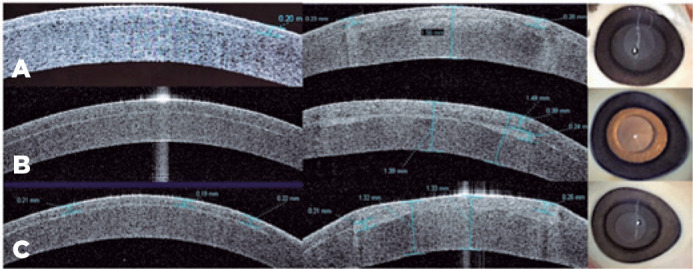
A, B, and C. Representative Visante® OCT images from groups A, B, and C, respectively, before (left) and after (middle) insertion of cornea, showing corneal flattening and gross appearance of the post-insertion corneas (right).

OCT images of the corneas from groups A, B, and C showed implants placed within the anterior stroma and gross flattening appearance, whereas the control group did not change shape (Figure 2). Gross photographs showed clear materials implanted within the corneal stroma (Figure 2).

The total final ablation depth deviated from the predicted depth. OCT was used to determine the average ablation depth at the center of the implants. For group A, the mean tissue ablated was 136 µm; group B, 150 µm; and group C, 240 µm, resulting in a 3-mm-diameter hole at the center of the material.

Based on the Munnerlyn formula for variable “t,” we used 136, 150, and 240 µm for groups A, B, and C, respectively. For all groups, we used a value of 5 for the variable “S.” Then, we estimated the mean final dioptric power of the lenticules as 16.4, 18.0, and 28.8 D for groups A, B, and C, respectively.

**Figure 3:**
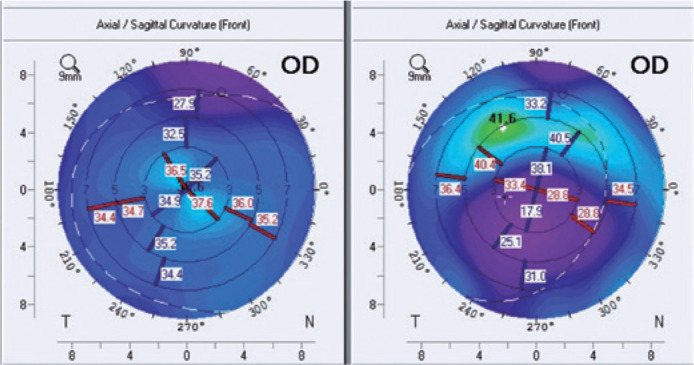
Representative Pentacam® images from a group A specimen before (left) and after (right) corneal insertion. A flatter shape was seen after biomaterial insertion.

Corneal tomography (Figure 3) indicated that the effect was decentered inferiorly, which could have arisen from suboptimal implant placement, alignment during imaging, or effect of the superior partial-thickness incision.

Corneal flattening was more evident in the groups with biomaterial implants, with a greater reduction of Km than the control group, although this reduction was not statistically significant. However, when comparing the pre-and post-Km among groups A, B, and C, this reduction approached significance (Table 1). Results of a separate comparison of K1 and K2 between the groups showed a statistically significant reduction of K1 (Table 1).

**Table 1:** Pentacam^***®***^ results (Km, K1, and K2) before and after implantation, comparing groups A-C (pooled data) with group D (control)

	Pre	Post	Delta (Pre-Post)	p1
**Km**				
**ABC**	35.16 (2.23)	31.64 (3.40)	3.52 (4.68)	0.066
**D**	33.78 (2.96)	33.12 (0.63)	0.67 (2.69)	1.000
p^^2^^	0.405	0.782	0.405	
**K1**				
**ABC**	34.04(2.78)	28.68 (3.16)	5.37 (4.89)	0.021^*^
**D**	31.70 (2.23)	32.07 (1.08)	-0.37 (2.05)	0.593
p^^2^^	0.229	0.096	0.116	
**K2**				
**ABC**	36.27 (1.94)	34.60 (4.52)	1.67 (4.91)	0.314
**D**	35.87 (3.93)	34.17 (0.60)	1.70 (3.35)	0.593
p^^2^^	0.644	0.782	0.926	

p^^1^^ - Wilcoxon matched-pairs signed-rank test.

p^^2^^ - Wilcoxon rank-sum test.

* statistically significant.

## DISCUSSION

Barraquer’s initial keratophakia experiments were conducted with different materials such as flint glass, plexiglass, semi-hydrated celloidin, and finally a lenticule of corneal tissue, as all previous experiments had failed. Barraquer suggested that the optimal material for inclusion in the cornea is the corneal parenchyma itself because of its physical (permeability and consistency), biological, and chemical characteristics^(5)^. Others have described the use of refractive corneal implants fashioned from synthetic materials or corneal tissues, including epikeratophakia^(6^,^16^,^17)^. In this study, we used a new biomaterial, a type I collagen-based vitrigel, which contains the main corneal structural component following what was suggested by Barraquer^(4^,^5)^. However, further studies are needed to evaluate the lenticule biomechanical properties.

The success of corneal reshaping with excimer laser encouraged research into laser preshaping of the lenticule (i.e., before implantation in the cornea). In 1991, Altmann et al. presented the first data on the use of the excimer laser for the creation of lenticules from human corneal tissues for epikeratoplasty in aphakic and myopic patients or lamellar corneal transplantation using a photoablation lathing process^(14)^. Altmann et al. reported choosing different parameters of the lenticule to set up the excimer laser corneal shaping system (ELCS-S) according to the indication, and the donor cornea was treated from the epithelial side in a holding device in front of a focused excimer laser beam. This group showed that ELCS-S is an alternative to cryolathe because of the unpredictable results of the freezing process^(14)^.

Ever since, several procedures have been designed around laser shaping of the lenticule. In 1999, Homolka et al. used an optimized scanning laser ablation algorithm with the excimer laser for highly accurate in vitro shaping of a refractive lenticule for use in corneal transplantation^(23)^. Later, Biowski et al. reported the results of an ELCS-S to produce human corneal tissue grafts to the surgeon’s precise specifications for lamellar transplantation^(24)^.

In 2002, Jankov et al. proposed the *laser-assisted intrastromal keratophakia* technique, which consisted of laser shaping of stromal implants from donor eyes, and reported good results in a human patient^(25)^. Two years later, the same authors implemented this technique to treat a patient with high hyperopia and irregular astigmatism secondary to multiple LASIK procedures^(12)^. Jankov et al. shaped the lenticule with PRK, and differently, the LASIK flap was relifted, the shaped lenticule was placed onto the stromal bed, and the flap was repositioned. The operation was uneventful as was the early postoperative follow-up^(12)^.

In 2018, Damgaard et al. described the benefits of PTK treatment of human donor lenticules, whether for the restoration of the corneal volume or for additive correction of refraction^(26)^. Zhao et al. reported the use of an autologous lenticule, shaped by small-incision lenticule extraction, in a patient with hyperopia after a LASIK flap complication^(15)^. Similar to Jankov’s^(12)^ data, the incomplete flap was peeled back superiorly to the hinge, a PTK was performed to decrease the central opacity, and the autologous lenticule was transplanted onto the stromal bed, without complication at 2 years follow-up^(15)^. The present study has a similar additive concept, but without the donor cornea. Such tissue also is unnecessary in the technique described by Chen et al., who used decellularized lenticules obtained from a small incision lenticule extraction (SMILE) as a new modality of corneal restoration^(27)^.

Mastropasqua et al. described the use of additive keratoplasty to treat advanced KC^(11)^. The pocket was performed by a femtosecond laser, enabling more precision than the manually dissected pocket used in the present experiment. However, to our knowledge, the present work is the first report demonstrating the potential feasibility of using an excimer laser-treated type I collagen-based implant for corneal reshaping. Such an approach could use topographic information, perhaps individualized, to shape the implant either during or after production.

The work described herein has limitations, such as the small sample size. However, our intent was to establish the feasibility of this line of research. Although the ablations produced in the implants were centered, the post-insertion topography appeared less so. A potential explanation could involve the decentration of the 6-mm implant within the 9-mm stromal pocket, which could be imprecise and variable in diameter because of the manual dissection technique used. The large incision made to initiate the pocket could also have affected the post-insertion topography, although we did not detect a flattening pattern in the axis of the incision as would have been expected. Another explanation could be a slight misalignment of the implanted eyes during Pentacam^®^ imaging. Additional limitations include the use of postmortem eyes, which present multiple technical challenges. A potential next step would be to study the biomaterials in an *in vivo* model.

In this study, a novel planoconcave-shaped biomaterial was used to flatten the cornea in a postmortem porcine eye model. This approach could be a novel treatment for refractive errors in eyes not amenable to currently available cornea-based treatment options, such as those with high myopia, thin corneas, corneal ectasia, or aphakia. However, much work is needed to further improve this approach for potential clinical applications.
